# Usefulness of tocilizumab for treating rheumatoid arthritis with myelodysplastic syndrome: A case report and literature review

**DOI:** 10.1097/MD.0000000000011179

**Published:** 2018-06-22

**Authors:** Chuanyin Sun, Yingwan Luo, Hongyan Tong, Guanhua Xu, Jin Lin

**Affiliations:** a Department of Rheumatology; b Department of Hematology, The First Affiliated Hospital, College of Medicine, Zhejiang University, Hangzhou, China.

**Keywords:** myelodysplastic syndrome, rheumatoid arthritis, tocilizumab

## Abstract

**Rationale:**

Dysregulated immune function in rheumatoid arthritis (RA) might lead to the development of myelodysplastic syndrome (MDS). Serum interleukin-6 (IL-6) concentrations are increased in both RA and MDS patients.

**Patient concerns:**

A 58-year-old woman presented with severe RA. During a recent 8-month period, the patient experienced swelling in multiple joints, dizziness, and severe anemia. The symptoms responded poorly to oral corticosteroids and methotrexate (MTX). Even treatment of the patient's anemia by transfusion of red blood cells was ineffective. Laboratory tests showed high levels of IL-6 (214.24 pg/mL).

**Diagnoses:**

Combining her medical history with clinical and laboratory parameters, especially those obtained by bone marrow aspiration, a diagnosis of RA with MDS was made.

**Interventions:**

MTX was discontinued and the patient was given tocilizumab intravenously at a dose of 8 mg/kg every 4 weeks and oral corticosteroids (15 mg/QD).

**Outcomes:**

The patient's serological, physical, and pathological abnormalities improved significantly.

**Lessons:**

We report a case of RA with MDS successfully treated with tocilizumab. To our knowledge, this is the first case of an RA patient with MDS that was successfully treated with tocilizumab. In addition, our case emphasizes that IL-6 plays a critical role in the pathogenesis of RA with MDS. Tocilizumab might be an effective treatment for RA with MDS, especially in those with high levels of IL-6, elevated C-reactive protein, and severe anemia.

## 1 Introduction

Rheumatoid arthritis (RA) is a common autoimmune disease, characterized by systemic inflammation and immunological high disability.^[1]^ Interleukin 6 (IL-6) is a pro-inflammatory cytokine and secreted by T cells and macrophages to stimulate immune response, and plays an important role in the development of RA.^[2]^ IL-6 aggravates the immune imbalance between regulatory T cells (Treg) and Th17 cells, and promotes the production of autoantibodies. IL-6 not only induces acute phase proteins including C-reaction protein (CRP), hepcidin, and fibrinogen, but also reduces the production of transferrin and albumin. High concentration of hepcidin blocked iron transporter 1, resulting in chronic inflammation and anemia.^[3]^

Myelodysplastic syndrome (MDS) is a group of heterogeneous acquired clonal diseases, resulting in the risk of ineffective hematopoiesis and malignant transformation.^[4]^ Some studies revealed a relationship between MDS and RA.^[5–7]^ In MDS patient, levels of serum inflammatory cytokines, such as IL-6 and tumor necrosis factor-a (TNF-a), are elevated compared to normal controls. In particular, IL-6 plays an important role in the progression of MDS. IL-6 is a co-stimulator of vitro bone marrow mesenchymal stem cells.^[3,8]^ We assume that anti-IL-6 monoclonal antibody (siltuximab) may have the potential to improve the prognosis of RA patients with MDS. We will present the case of a patient with RA and MDS who was successfully treated with tocilizumab.

## 2 Case report

**Figure 1:**
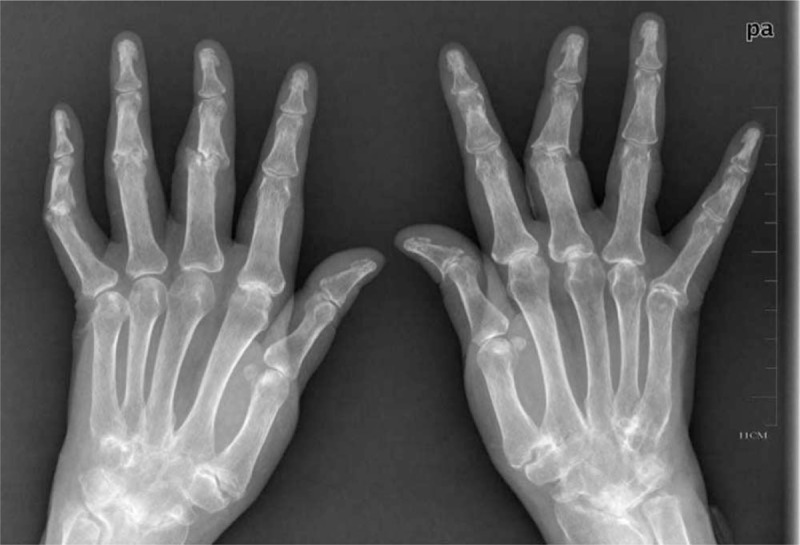
Radiologic erosions. Both hand x-ray images demonstrated advanced arthritis combined with body ankylosis.

A 58-year-old woman with a 2-year history of polyarthropathy had a diagnosis of RA (Fig. 1). She was treated with oral corticosteroids (15 mg/QD), methotrexate (MTX) 10 mg weekly, and/or a nonsteroidal antiinflammatory drug. Her steady situation lasted for 16 months. In the recent 8 months, she experienced severely impairing and dizzines s and anemia. The blood test revealed normocytic anemia and she was admitted to the local hospital. Laboratory results were as follows: white blood cell (WBC) count, 5.48 × 10^9^ cells/L; hemoglobin (Hb) count, 34 g/L; erythrocyte mean corpuscular volume (MCV), 89.0 fl, platelet (PLT) count, 381 × 10^9^ cells/L; erythrocyte sedimentation rate (ESR), 138 mm/h; CRP, 117 mg/L; rheumatoid factor, 223 IU/mL; anti-cyclic peptide containing citrulline, 885.6 RU/mL; anti-nuclear antibody/anti-phospholipid antibodies/anti-neutrophil cytoplasmic antibodies, negative; vitamin B12, 368 pmol/L; folic acid, 4.6 nmol/L; erythropoietin, normal; serum ferritin, 287.96 ng/mL. At this point, MTX was discontinued and the patient received red blood cell transfusions. After treatment with glucocorticoids (methylprednisolone 4 mg/TID for 12 weeks), joint symptoms and CRP/ESR improved. However, the patient's hemoglobin level declined to 32 g/L. Even though folic acid tablets and ferrous sulfate were also administered, the response remained poor. The patient was referred to our hospital. Re-examination was conducted after red blood cell transfusion with the following laboratory data: WBC, 2.3 × 10^9^ cells/L; Hb, 49 g/L; PLT, 237 × 10^9^ cells/L; ESR, 108 mm/h; CRP, 61 mg/L; ferritin, 2325 g/L; and IL-6, 214.24 pg/mL.

**Figure 2:**
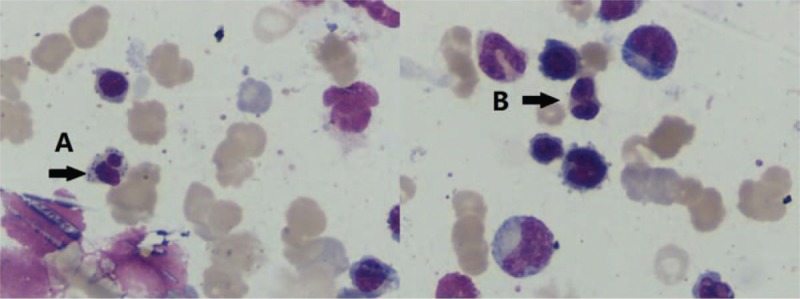
Bone marrow aspirate of the rheumatoid arthritis/myelodysplastic syndrome patient. (A) Erythrocyte basophilic stippling. (B) Double nuclei in metamyelocyte.

The examination of bone marrow aspiration demonstrated dysplastic features on 2 hematopoietic lineages, but more prominent in the erythroid, which showed clustering with nuclear budding, pedal nuclei, and H-J bodies (dysplasis >10%). The double nuclei and megaloblastic changes could also be observed in the granulocytic lineage (accounting for 4%). The number of megakaryocytes was increased and the production of thrombocytes was fine (Fig. 2). In addition, bone marrow biopsy also revealed hypercellularity and erythroid hyperplasia. Cytogenetic analysis showed a normal 46 XX [20] karyotype.

Combining her medical history with the clinical and laboratory parameters, a diagnosis of treatment-related MDS (t-MDS) was made. Disease modifying antirheumatic drug (MTX) might have been responsible for the bone marrow toxicity.

These results, in combination with the patient's history, led to the addition of tocilizumab to the treatment regimen. To evaluate the clinical effects of tocilizumab in the RA patient, WBC, Hb, ferritin, ESR/CRP, and IL-6 were assessed at baseline, 1st, 3rd, and 6th months of tocilizumab treatment. She started therapy with intravenous injection of tocilizumab (8 mg/kg) every 4 weeks and oral corticosteroids (15 mg/QD), achieving clinical benefit and improvement in symptoms. One month after admission, the patient was discharged from hospital. Laboratory results at that time were: WBC, 8.6 × 10^9^ cells/L; Hb, 97 g/L; CRP, 7.2 mg/L; ESR, 26 mm/h; ferritin, 936 g/L; and IL-6, 23.85 pg/mL. Upon hospital admission, 2 months after the regular checkup, the results were: WBC, 6.9 × 10^9^ cells/L; Hb, 73 g/L; CRP, 41 mg/L; ESR, 38 mm/h; ferritin, 231 g/L; and IL-6, 1.12 pg/mL. Repeat bone marrow aspiration at the 6-month follow-up showed that dysplasia was not obvious. Laboratory results showed no obvious abnormality. This resulted in the prompt resolution of the patient's serological, physical, and pathological abnormalities. Hence, the response to tocilizumab treatment was very good.

**Figure 3:**
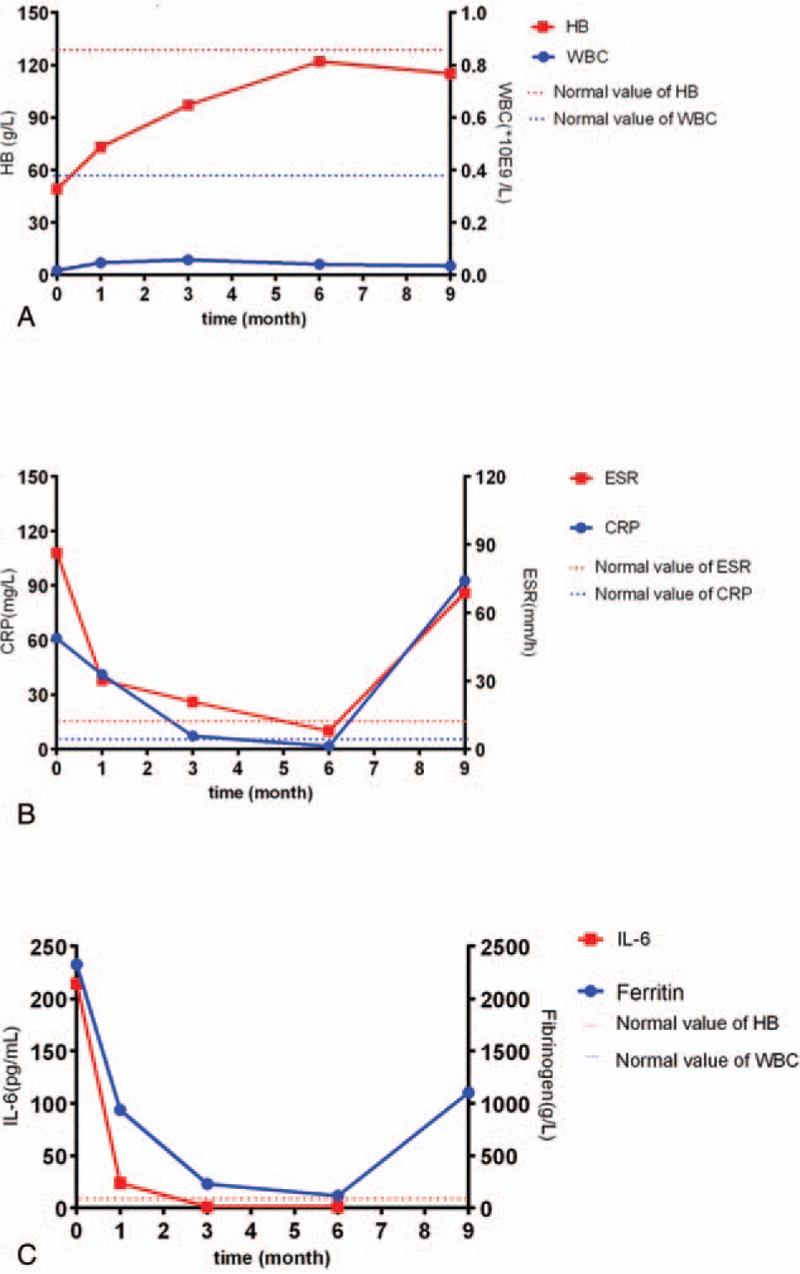
Laboratory changes during the clinical course.

However, it was shown to be unlikely that the use of low doses of steroids in the patient was responsible for the sustained disease control. The patient refused the injection of tocilizumab after the 6th month. CRP (92.6 mg/L) increased and joint pain returned. Despite this relapse, the patient's laboratory examination was stable and symptoms significantly improved after resuming treatment with tocilizumab (Fig. 3).

## 3 Discussion

MDS is a common hematologic disease that is characterized by ineffective hematopoiesis and a tendency to progress into acute myeloid leukemia.^[4]^ Accumulating evidence suggests that immune mechanisms involve bone marrow failure in MDS. Autoimmune-related complications in MDS may be more common than previously thought.^[9,10]^ A few reported cases of RA associated with MDS suggested that dysregulated immune function in RA might lead to the development of MDS.^[11,12]^ We deem that this fundamental change in the state of the RA was conditioned by the MDS and its consequences on the imbalanced immune system.

Therapy-related MDS has also been reported in cases with connective tissue diseases.^[13]^ Although rarely described, MDS has been reported in patients with RA after MTX therapy.^[14–17]^ Thonhofer describes significant myelodysplastic changes in RA patients, suggesting that this change may be caused by an inflammatory microenvironment in active RA.^[14]^ Although MTX provides good efficacy in RA patients, the rapid progress of MDS in our patient while continuing MTX suggests that MTX should be discontinued once MDS is confirmed in RA patients.

It has been shown that the development of RA is strongly associated with IL-6.^[2]^ IL-6 has been reported to induce hepcidin, which is a key mediator of anemia in inflammation through its inhibition of iron excretion protein ferritin results in the retention of iron in cells. Abnormal iron accumulation is common in MDS. Elevated levels of IL-6 have been negatively correlated with anemia.^[9]^ IL-6 may play an important role in the progression of MDS. IL-6 is a co-stimulator of vitro bone marrow mesenchymal stem cells.^[18]^ Since IL-6 levels are significantly higher in MDS marrow than in comparable cells from healthy controls and the serum IL-6 concentrations are increased in RA patients, IL-6 antagonist (tocilizumab) would theoretically be ideal choice for the treatment of RA patients with MDS.^[19]^ Therefore, we selected the therapy for RA with MDS and expected that tocilizumab simultaneously treat both inflammation and hematopoietic abnormality in this patient. As a matter of fact, these symptoms and abnormalities were improved by the treatment. Here we present the possibility that tocilizumab might be an effective treatment for RA with MDS, especially in those with high levels of IL-6, elevated CRP, and severe anemia.

In a double-blind, placebo-controlled Phase II study, anti-IL-6 monoclonal antibody (siltuximab) was assessed in patients with low- and intermediate-1-risk MDS who require regularly transfusions for MDS anemia. However, siltuximab therapy was not effective in reducing red blood cell transfusions in MDS patients compared to placebo.^[20]^ However, siltuximab therapy responded well in the cases with elevated inflammation, leading to amelioration of the anemia.^[21]^

Immunosuppressive therapy can induce hematological recovery in MDS patients. Improvement of cytopenias in MDS patients after cortisol treatment has been described. A previous case of RA with MDS was also successfully treated with corticosteroids.^[16,17]^ However, in RA patients with MDS refractory to corticosteroids, the use of IL-6 monoclonal antibodies could be considered. Moreover, many physicians may avoid using a high dose of corticosteroids in elderly patients. In this case, we selected tocilizumab as an alternative to increasing the dose of corticosteroids.

In conclusion, we recommend anti-IL-6 therapy in patients with RA comorbid with MDS, although more data regarding the efficacy and safety of anti-IL-6 antibodies are needed. We considered that the long-term improvement in hematopoietic abnormality was caused by the effect of combination therapy with tocilizumab and corticosteroids in this patient. This is the first report of a patient with RA complicated by MDS that was successfully treated with tocilizumab, an IL-6 antagonist. Tocilizumab distinctly improved the patient's symptom and cytopenias. Clinical trials are still needed to verify the efficacy of tocilizumab on RA patients with MDS.

## Author contributions

**Data curation:** Yingwan Luo, Guanhua Xu.

**Supervision:** Hongyan Tong.

**Writing – original draft:** Chuanyin Sun.

**Writing – review & editing:** Jin Lin.
